# Supplementary material for: A Critical View of the Application of the APEX Software (Aqueous Photochemistry of Environmentally-Occurring Xenobiotics) to Predict Photoreaction Kinetics in Surface Freshwaters
Source: Molecules. 2019 Dec 18;25(1):9. doi: 10.3390/molecules25010009 (PMC7017383; doi:10.3390/molecules25010009)
Supplement: Supplementary file 1 [file molecules-25-00009-s001.zip › 3.2.4_gcc-4.4.0.zip!/3.2.4_gcc-4.4.0/3.2.4_gcc-4.4.0/doc/octave/html/interpreter/Sparse-Linear-Algebra.html]

Sparse Linear Algebra - Untitled


Next: Iterative Techniques,
Previous: Basics,
Up: Sparse Matrices


---

### 21.2 Linear Algebra on Sparse Matrices

Octave includes a polymorphic solver for sparse matrices, where
the exact solver used to factorize the matrix, depends on the properties
of the sparse matrix itself. Generally, the cost of determining the matrix type
is small relative to the cost of factorizing the matrix itself, but in any
case the matrix type is cached once it is calculated, so that it is not
re-determined each time it is used in a linear equation.

The selection tree for how the linear equation is solve is

1. If the matrix is diagonal, solve directly and goto 8- If the matrix is a permuted diagonal, solve directly taking into
     account the permutations. Goto 8- If the matrix is square, banded and if the band density is less
       than that given by `spparms ("bandden")` continue, else goto 4.
       1. If the matrix is tridiagonal and the right-hand side is not sparse
          continue, else goto 3b.
          1. If the matrix is hermitian, with a positive real diagonal, attempt
             Cholesky factorization using lapack xPTSV.- If the above failed or the matrix is not hermitian with a positive
               real diagonal use Gaussian elimination with pivoting using
               lapack xGTSV, and goto 8.
          - If the matrix is hermitian with a positive real diagonal, attempt
            Cholesky factorization using lapack xPBTRF.- if the above failed or the matrix is not hermitian with a positive
              real diagonal use Gaussian elimination with pivoting using
              lapack xGBTRF, and goto 8.
       - If the matrix is upper or lower triangular perform a sparse forward
         or backward substitution, and goto 8- If the matrix is a upper triangular matrix with column permutations
           or lower triangular matrix with row permutations, perform a sparse forward
           or backward substitution, and goto 8- If the matrix is square, hermitian with a real positive diagonal, attempt
             sparse Cholesky factorization using CHOLMOD.- If the sparse Cholesky factorization failed or the matrix is not
               hermitian with a real positive diagonal, and the matrix is square, factorize
               using UMFPACK.- If the matrix is not square, or any of the previous solvers flags
                 a singular or near singular matrix, find a minimum norm solution using
                 CXSPARSE^1^.

The band density is defined as the number of non-zero values in the matrix
divided by the number of non-zero values in the matrix. The banded matrix
solvers can be entirely disabled by using spparms to set `bandden`
to 1 (i.e., `spparms ("bandden", 1)`).

The QR solver factorizes the problem with a Dulmage-Mendelsohn, to
separate the problem into blocks that can be treated as over-determined,
multiple well determined blocks, and a final over-determined block. For
matrices with blocks of strongly connected nodes this is a big win as
LU decomposition can be used for many blocks. It also significantly
improves the chance of finding a solution to over-determined problems
rather than just returning a vector of NaN's.

All of the solvers above, can calculate an estimate of the condition
number. This can be used to detect numerical stability problems in the
solution and force a minimum norm solution to be used. However, for
narrow banded, triangular or diagonal matrices, the cost of
calculating the condition number is significant, and can in fact
exceed the cost of factoring the matrix. Therefore the condition
number is not calculated in these cases, and Octave relies on simpler
techniques to detect singular matrices or the underlying lapack code in
the case of banded matrices.

The user can force the type of the matrix with the `matrix_type`
function. This overcomes the cost of discovering the type of the matrix.
However, it should be noted that identifying the type of the matrix incorrectly
will lead to unpredictable results, and so `matrix_type` should be
used with care.

— Function File: [n, c] = **normest** (a, tol)  
> Estimate the 2-norm of the matrix a using a power series
> analysis. This is typically used for large matrices, where the cost
> of calculating the `norm (`a`)` is prohibitive and an approximation
> to the 2-norm is acceptable.
>
> tol is the tolerance to which the 2-norm is calculated. By default
> tol is 1e-6. c returns the number of iterations needed for
> `normest` to converge.

— Function File: [est, v, w, iter] = **onenormest** (a, t)  
— Function File: [est, v, w, iter] = **onenormest** (apply, apply\_t, n, t)  
> Apply Higham and Tisseur's randomized block 1-norm estimator to
> matrix a using t test vectors. If t exceeds 5, then
> only 5 test vectors are used.
>
> If the matrix is not explicit, e.g., when estimating the norm of
> `inv (`A`)` given an LU factorization, `onenormest` applies
> A and its conjugate transpose through a pair of functions
> apply and apply\_t, respectively, to a dense matrix of size
> n by t. The implicit version requires an explicit dimension
> n.
>
> Returns the norm estimate est, two vectors v and
> w related by norm
> `(`w`, 1) =` est `* norm (`v`, 1)`,
> and the number of iterations iter. The number of
> iterations is limited to 10 and is at least 2.
>
> References:
>
> - Nicholas J. Higham and Françoise Tisseur, "A Block Algorithm
>   for Matrix 1-Norm Estimation, with an Application to 1-Norm
>   Pseudospectra." SIMAX vol 21, no 4, pp 1185-1201.
>   http://dx.doi.org/10.1137/S0895479899356080- Nicholas J. Higham and Françoise Tisseur, "A Block Algorithm
>     for Matrix 1-Norm Estimation, with an Application to 1-Norm
>     Pseudospectra." http://citeseer.ist.psu.edu/223007.html
>
>
>
>
> **See also:** condest, norm, cond.

— Function File: [est, v] = **condest** (a, t)  
— Function File: [est, v] = **condest** (a, solve, solve\_t, t)  
— Function File: [est, v] = **condest** (apply, apply\_t, solve, solve\_t, n, t)  
> Estimate the 1-norm condition number of a matrix A
> using t test vectors using a randomized 1-norm estimator.
> If t exceeds 5, then only 5 test vectors are used.
>
> If the matrix is not explicit, e.g., when estimating the condition
> number of a given an LU factorization, `condest` uses the
> following functions:
>
> apply: `A*x` for a matrix `x` of size n by t. apply\_t: `A'*x` for a matrix `x` of size n by t. solve: `A \ b` for a matrix `b` of size n by t. solve\_t: `A' \ b` for a matrix `b` of size n by t.
>
> The implicit version requires an explicit dimension n.
>
> `condest` uses a randomized algorithm to approximate
> the 1-norms.
>
> `condest` returns the 1-norm condition estimate est and
> a vector v satisfying `norm (A*v, 1) == norm (A, 1) * norm
> (`v`, 1) /` est. When est is large, v is an
> approximate null vector.
>
> References:
>
> - Nicholas J. Higham and Françoise Tisseur, "A Block Algorithm
>   for Matrix 1-Norm Estimation, with an Application to 1-Norm
>   Pseudospectra." SIMAX vol 21, no 4, pp 1185-1201.
>   http://dx.doi.org/10.1137/S0895479899356080- Nicholas J. Higham and Françoise Tisseur, "A Block Algorithm
>     for Matrix 1-Norm Estimation, with an Application to 1-Norm
>     Pseudospectra." http://citeseer.ist.psu.edu/223007.html
>
>
>
>
> **See also:** cond, norm, onenormest.

— Loadable Function: **spparms** ()  
— Loadable Function: vals = **spparms** ()  
— Loadable Function: [keys, vals] = **spparms** ()  
— Loadable Function: val = **spparms** (key)  
— Loadable Function: **spparms** (vals)  
— Loadable Function: **spparms** ('defaults')  
— Loadable Function: **spparms** ('tight')  
— Loadable Function: **spparms** (key, val)  
> Sets or displays the parameters used by the sparse solvers and factorization
> functions. The first four calls above get information about the current
> settings, while the others change the current settings. The parameters are
> stored as pairs of keys and values, where the values are all floats and the
> keys are one of the following strings:
>
> `spumoni`: Printing level of debugging information of the solvers (default 0) `ths_rel`: Included for compatibility. Not used. (default 1) `ths_abs`: Included for compatibility. Not used. (default 1) `exact_d`: Included for compatibility. Not used. (default 0) `supernd`: Included for compatibility. Not used. (default 3) `rreduce`: Included for compatibility. Not used. (default 3) `wh_frac`: Included for compatibility. Not used. (default 0.5) `autommd`: Flag whether the LU/QR and the '\' and '/' operators will automatically use the sparsity preserving mmd functions (default 1) `autoamd`: Flag whether the LU and the '\' and '/' operators will automatically use the sparsity preserving amd functions (default 1) `piv_tol`: The pivot tolerance of the UMFPACK solvers (default 0.1) `sym_tol`: The pivot tolerance of the UMFPACK symmetric solvers (default 0.001) `bandden`: The density of non-zero elements in a banded matrix before it is treated by the lapack banded solvers (default 0.5) `umfpack`: Flag whether the UMFPACK or mmd solvers are used for the LU, '\' and '/' operations (default 1)
>
> The value of individual keys can be set with `spparms (`key`,` val`)`. The default values can be restored with the special keyword
> 'defaults'. The special keyword 'tight' can be used to set the mmd solvers
> to attempt for a sparser solution at the potential cost of longer running
> time.

— Loadable Function: p = **sprank** (s)  
> Calculates the structural rank of a sparse matrix s. Note that
> only the structure of the matrix is used in this calculation based on
> a Dulmage-Mendelsohn permutation to block triangular form. As such the numerical
> rank of the matrix s is bounded by `sprank (`s`) >=
> rank (`s`)`. Ignoring floating point errors `sprank (`s`) ==
> rank (`s`)`.
>
>
>
> **See also:** dmperm.

— Loadable Function: [count, h, parent, post, r] = **symbfact** (s, typ, mode)  
> Performs a symbolic factorization analysis on the sparse matrix s.
> Where
>
> s: s is a complex or real sparse matrix. typ: Is the type of the factorization and can be one of `sym`: Factorize s. This is the default. `col`: Factorize s`' *` s. `row`: Factorize s `*` s`'`. `lo`: Factorize s`'` mode: The default is to return the Cholesky factorization for r, and if mode is 'L', the conjugate transpose of the Cholesky factorization is returned. The conjugate transpose version is faster and uses less memory, but returns the same values for count, h, parent and post outputs.
>
> The output variables are
>
> count: The row counts of the Cholesky factorization as determined by typ. h: The height of the elimination tree. parent: The elimination tree itself. post: A sparse boolean matrix whose structure is that of the Cholesky factorization as determined by typ.

For non square matrices, the user can also utilize the `spaugment`
function to find a least squares solution to a linear equation.

— Function File: s = **spaugment** (a, c)  
> Creates the augmented matrix of a. This is given by
>
> ```
>           [c * eye(m, m),a; a', zeros(n,
>           n)]
> ```
>
> This is related to the least squares solution of
> a `\\` b, by
>
> ```
>           s * [ r / c; x] = [b, zeros(n,
>           columns(b)]
> ```
>
> where r is the residual error
>
> ```
>           r = b - a * x
> ```
>
> As the matrix s is symmetric indefinite it can be factorized
> with `lu`, and the minimum norm solution can therefore be found
> without the need for a `qr` factorization. As the residual
> error will be `zeros (`m`,` m`)` for under determined
> problems, and example can be
>
> ```
>           m = 11; n = 10; mn = max(m ,n);
>           a = spdiags ([ones(mn,1), 10*ones(mn,1), -ones(mn,1)],
>                        [-1, 0, 1], m, n);
>           x0 = a \ ones (m,1);
>           s = spaugment (a);
>           [L, U, P, Q] = lu (s);
>           x1 = Q * (U \ (L \ (P  * [ones(m,1); zeros(n,1)])));
>           x1 = x1(end - n + 1 : end);
> ```
>
> To find the solution of an overdetermined problem needs an estimate
> of the residual error r and so it is more complex to formulate
> a minimum norm solution using the `spaugment` function.
>
> In general the left division operator is more stable and faster than
> using the `spaugment` function.

Finally, the function `eigs` can be used to calculate a limited
number of eigenvalues and eigenvectors based on a selection criteria
and likewise for `svds` which calculates a limited number of
singular values and vectors.

— Loadable Function: d **=** eigs (a)  
— Loadable Function: d **=** eigs (a, k)  
— Loadable Function: d **=** eigs (a, k, sigma)  
— Loadable Function: d **=** eigs (a, k, sigma,opts)  
— Loadable Function: d **=** eigs (a, b)  
— Loadable Function: d **=** eigs (a, b, k)  
— Loadable Function: d **=** eigs (a, b, k, sigma)  
— Loadable Function: d **=** eigs (a, b, k, sigma, opts)  
— Loadable Function: d **=** eigs (af, n)  
— Loadable Function: d **=** eigs (af, n, b)  
— Loadable Function: d **=** eigs (af, n, k)  
— Loadable Function: d **=** eigs (af, n, b, k)  
— Loadable Function: d **=** eigs (af, n, k, sigma)  
— Loadable Function: d **=** eigs (af, n, b, k, sigma)  
— Loadable Function: d **=** eigs (af, n, k, sigma, opts)  
— Loadable Function: d **=** eigs (af, n, b, k, sigma, opts)  
— Loadable Function: [v, d] **=** eigs (a, ...)  
— Loadable Function: [v, d] **=** eigs (af, n, ...)  
— Loadable Function: [v, d, flag] **=** eigs (a, ...)  
— Loadable Function: [v, d, flag] **=** eigs (af, n, ...)  
> Calculate a limited number of eigenvalues and eigenvectors of a,
> based on a selection criteria. The number eigenvalues and eigenvectors to
> calculate is given by k whose default value is 6.
>
> By default `eigs` solve the equation
> `A * v = lambda * v`
> , where
> `lambda` is a scalar representing one of the eigenvalues, and `v`
> is the corresponding eigenvector. If given the positive definite matrix
> B then `eigs` solves the general eigenvalue equation
> `A * v = lambda * B * v`
> .
>
> The argument sigma determines which eigenvalues are returned.
> sigma can be either a scalar or a string. When sigma is a scalar,
> the k eigenvalues closest to sigma are returned. If sigma
> is a string, it must have one of the values
>
> 'lm': Largest magnitude (default). 'sm': Smallest magnitude. 'la': Largest Algebraic (valid only for real symmetric problems). 'sa': Smallest Algebraic (valid only for real symmetric problems). 'be': Both ends, with one more from the high-end if k is odd (valid only for real symmetric problems). 'lr': Largest real part (valid only for complex or unsymmetric problems). 'sr': Smallest real part (valid only for complex or unsymmetric problems). 'li': Largest imaginary part (valid only for complex or unsymmetric problems). 'si': Smallest imaginary part (valid only for complex or unsymmetric problems).
>
> If opts is given, it is a structure defining some of the options that
> `eigs` should use. The fields of the structure opts are
>
> `issym`: If af is given, then flags whether the function af defines a symmetric problem. It is ignored if a is given. The default is false. `isreal`: If af is given, then flags whether the function af defines a real problem. It is ignored if a is given. The default is true. `tol`: Defines the required convergence tolerance, given as `tol * norm (A)`. The default is `eps`. `maxit`: The maximum number of iterations. The default is 300. `p`: The number of Lanzcos basis vectors to use. More vectors will result in faster convergence, but a larger amount of memory. The optimal value of 'p' is problem dependent and should be in the range k to n. The default value is `2 *` k. `v0`: The starting vector for the computation. The default is to have Arpack randomly generate a starting vector. `disp`: The level of diagnostic printout. If `disp` is 0 then there is no printout. The default value is 1. `cholB`: Flag if `chol (`b`)` is passed rather than b. The default is false. `permB`: The permutation vector of the Cholesky factorization of b if `cholB` is true. That is `chol (` b `(permB, permB))`. The default is `1:`n.
>
> It is also possible to represent a by a function denoted af.
> af must be followed by a scalar argument n defining the length
> of the vector argument accepted by af. af can be passed either
> as an inline function, function handle or as a string. In the case where
> af is passed as a string, the name of the string defines the function
> to use.
>
> af is a function of the form `function y = af (x), y = ...;
> endfunction`, where the required return value of af is determined by
> the value of sigma, and are
>
> `A * x`: If sigma is not given or is a string other than 'sm'. `A \ x`: If sigma is 'sm'. `(A - sigma * I) \ x`: for standard eigenvalue problem, where `I` is the identity matrix of the same size as `A`. If sigma is zero, this reduces the `A \ x`. `(A - sigma * B) \ x`: for the general eigenvalue problem.
>
> The return arguments of `eigs` depends on the number of return
> arguments. With a single return argument, a vector d of length k
> is returned, represent the k eigenvalues that have been found. With two
> return arguments, v is a n-by-k matrix whose columns are
> the k eigenvectors corresponding to the returned eigenvalues. The
> eigenvalues themselves are then returned in d in the form of a
> n-by-k matrix, where the elements on the diagonal are the
> eigenvalues.
>
> Given a third return argument flag, `eigs` also returns the status
> of the convergence. If flag is 0, then all eigenvalues have converged,
> otherwise not.
>
> This function is based on the Arpack package, written by R Lehoucq,
> K Maschhoff, D Sorensen and C Yang. For more information see
> http://www.caam.rice.edu/software/ARPACK/.


**See also:** eig, svds.

— Function File: s = **svds** (a)  
— Function File: s = **svds** (a, k)  
— Function File: s = **svds** (a, k, sigma)  
— Function File: s = **svds** (a, k, sigma, opts)  
— Function File: [u, s, v, flag] = **svds** (...)  
> Find a few singular values of the matrix a. The singular values
> are calculated using
>
> ```
>           [m, n] = size(a)
>           s = eigs([sparse(m, m), a; ...
>                           a', sparse(n, n)])
> ```
>
> The eigenvalues returned by `eigs` correspond to the singular
> values of a. The number of singular values to calculate is given
> by k, whose default value is 6.
>
> The argument sigma can be used to specify which singular values
> to find. sigma can be either the string 'L', the default, in
> which case the largest singular values of a are found. Otherwise
> sigma should be a real scalar, in which case the singular values
> closest to sigma are found. Note that for relatively small values
> of sigma, there is the chance that the requested number of singular
> values are not returned. In that case sigma should be increased.
>
> If opts is given, then it is a structure that defines options
> that `svds` will pass to eigs. The possible fields of this
> structure are therefore determined by `eigs`. By default three
> fields of this structure are set by `svds`.
>
> `tol`: The required convergence tolerance for the singular values. `eigs` is passed tol divided by `sqrt(2)`. The default value is 1e-10. `maxit`: The maximum number of iterations. The default is 300. `disp`: The level of diagnostic printout. If `disp` is 0 then there is no printout. The default value is 0.
>
> If more than one output argument is given, then `svds` also
> calculates the left and right singular vectors of a. flag
> is used to signal the convergence of `svds`. If `svds`
> converges to the desired tolerance, then flag given by
>
> ```
>           norm (a * v - u * s, 1) <= ...
>                   tol * norm (a, 1)
> ```
>
> will be zero.


**See also:** eigs.

---

#### Footnotes

[1] The CHOLMOD, UMFPACK and CXSPARSE packages were
written by Tim Davis and are available at
http://www.cise.ufl.edu/research/sparse/

---
